# Changes in Cadmium Concentration in Muscles, Ovaries, and Eggs of Silver European Eel (*Anguilla anguilla*) during Maturation under Controlled Conditions

**DOI:** 10.3390/ani11041027

**Published:** 2021-04-05

**Authors:** Joanna Nowosad, Dariusz Kucharczyk, Mariusz Szmyt, Joanna Łuczynska, Müller Tamás, László Horváth

**Affiliations:** 1 Department of Ichthyology and Aquaculture, Faculty of Animal Bioengineering, University of Warmia and Mazury, 10-719 Olsztyn, Poland; nowosad.joanna@gmail.com (J.N.); mariusz.szmyt@uwm.edu.pl (M.S.); 2 ChemProf, 11-041 Olsztyn, Poland; 3 Department of Commodity and Food Analysis, University of Warmia and Mazury, 10-726 Olsztyn, Poland; jlucz@uwm.edu.pl; 4 Department of Freshwater Fish Ecology, Hungarian University of Agricultural and Life Sciences, 2100 Gödöllő, Hungary; muller.tamas@mkk.szie.hu; 5 Department of Aquaculture, Hungarian University of Agricultural and Life Sciences, 2100 Gödöllő, Hungary; horvath.laszlo.matyas@gmail.com

**Keywords:** cadmium, European eel, ovary, eggs, bioaccumulation

## Abstract

**Simple Summary:**

The European eel (*Anguilla anguilla*) is currently a critically endangered fish species (IUCN list). This is thought to be due to many factors, including pollution of the aquatic environment with heavy metals, especially cadmium (Cd) and mercury (Hg), which may have a negative impact on the condition and abundance of the European eel population. Owing to bioaccumulation, the level of cadmium in the tissues of fish, especially those at the top of the trophic chain, may be significant and endanger the health and effective spawning success of the species due to impairment of the reproductive capacity. In this study, it was found that female eels’ muscles contain significantly less cadmium than their ovaries and eggs. This indicates the transfer of cadmium from the maternal organism to the offspring organism.

**Abstract:**

This study determined the contents of cadmium (Cd) in the muscles, ovaries, and eggs of silver female European eels. The analysis of cadmium content was performed on female European eels caught during commercial fishing in freshwater in Warmia and Mazury (Poland), and then subjected to artificial maturation and ovulation processing under controlled conditions. The content of cadmium (Cd) in the tissues was determined by flameless atomic spectrometry using an electrothermal atomizer. The analysis showed statistically significant differences between the cadmium content in the muscles, ovaries, and eggs (*p* < 0.05) of female European eels. The lowest cadmium content was found in the muscle tissue (0.0012 ± 0.0001 mg kg^−1^ wet weight) and the highest in eggs (after ovulation) (0.0038 ± 0.0007 mg kg^−1^ wet weight). Moreover, a relationship was found between the cadmium content in the muscle tissue and the ovaries (*R* = 0.673; *p* = 0.0117) in the same fish. The movement of cadmium from tissues to oocytes may indicate a significant problem concerning this heavy metal content in the reproduction of European eel.

## 1. Introduction

The progressive development of civilization and modern technology has brought many benefits and conveniences to people. Unfortunately, the development of civilization also has a negative impact, especially on the natural environment. It results in continuous and often irreversible changes in the environment, which directly or indirectly negatively affect the animals living in it. Organisms at the top of the trophic chain are the most exposed to environmental pollution [1,2,3]. Increasing amounts of discharged industrial waste, household waste, or run-off from agricultural land flowing into the aquatic environment may have a negative impact on aquatic organisms and the human population [1,2,4]. Pollution of the aquatic ecosystem by oil derivatives, pesticides, microplastics, heavy metals, and so on can lead to their accumulation in both water and fish tissues. Currently, many fish populations are endangered owing to the adverse effects of pollution in the natural environment. Fish tend to bioaccumulate these chemicals and are then consumed by humans at the top of the food chain [3,5,6,7,8].

Currently, owing to high toxicity and the fact that they are endocrine-disrupting chemicals (EDCs), much research has been conducted on the pollution of the environment and animals with dioxins and heavy metals, e.g., mercury (Hg), lead (Pb), and cadmium (Cd) [4,9,10]. A major role in spreading pollution is played by waters in which they are accumulated [7]. Heavy metals, such as Hg or Cd, do not often play any biological function and can be harmful to living organisms, even in small amounts [11]. They persist in the environment, which allows their bioaccumulation in water, deposits, and organic tissues [2,12]. Cadmium is regarded as one of the most hazardous heavy metals [2,13], as it is easily absorbed and persists for a long time in tissues, where it is bioaccumulated. Cadmium concentrations in tissues of wild fish, especially those closer to the top of the trophic chain, can be quite high and they can be harmful not only to the fish health, but also to the effective continuation of the species as a result of reproductive function disorders [12,14,15,16]. The eel is a critically endangered fish species with high susceptibility to heavy metals and organic pollutants (like polychlorinated biphenyls (PCBs), dichlorodiphenyltrichloroethane (DDT), or their breakdown products) exposure [17,18,19].

The European eel is a catadromous species, which migrates for spawning from freshwaters to salty waters in the Sargasso Sea [20]. In the late 20th century, the population of this fish declined by about 90% [21] and the species was added to the list of critically endangered animals [22]. This has been attributed to overfishing, obstacles the fish encounter on their migration routes (weirs, dams), infestation with the nematode *Anguillcoloides crassus*, as well as high levels of contamination accumulated in the animals [18,22]. Developing methods for artificial reproduction [23,24,25,26] and regular stocking [27] is among the ways of preserving the European eel in the natural environment. As the maturation process in the eel is much longer (e.g., [26,28]) than in other fish species (e.g., [29,30]), lasting up to several dozen weeks in eel [31,32,33], it requires comprehensive studies [26,32,33,34,35]. This can be attributed to the fact that the eels’ environment changes from freshwater to saltwater during their spawning journey [31]. Eels stop feeding during the migration [36], and they use energy accumulated in freshwater for building their gonads. According to the findings of the study conducted by Baeza et al. [34] and Nowosad et al. [33], gonad maturation is accompanied by migrations of proteins and lipids within the body. Unfortunately, this can also apply to heavy metals accumulated in the spawners’ bodies [37,38].

As not all chemical pollutants can be bioavailable in equal quantities and different pollutants can bioaccumulate in living organisms to a greater extent than others, the levels of pollutants in the body must be investigated to determine the risks arising from exposure to such pollutants in the environment [39]. Therefore, chemical analysis of tissues of aquatic organisms, such as fish, is used as a routine model in studies to assess the aquatic environment pollution, thus ensuring timely assessment of the levels of biological pollutants at higher concentrations in sediment or water and enabling their quantification [39]. Determination of the cadmium content in muscles, ovaries, and eggs may, in future, allow for tracing the metabolic pathways of this heavy metal between the tissues of the mother and the offspring. Moreover, the relationship between protein and fat and cadmium levels in the examined tissues was determined.

This study aimed to determine the content of cadmium in muscles, gonads, and eggs of silver female European eel during the period of maturation under controlled conditions. Moreover, the relationships between fish weight and length and cadmium content in muscle and ovary, between cadmium content in muscle and ovary, and between protein and fat content and cadmium content in muscle and ovary were investigated.

## 2. Materials and Methods

### 2.1. Fish

Migrating female European eels (silver form) with an average body weight of 1063.80 ± 356.38 g and body length of 82.3 ± 9.1 cm (mean ± SD) were caught during commercial fishing activities in freshwater lakes in the Warmia and Mazury region, connected with the Sawica river (the north-east of Poland), from the Baltic Sea basin. The place where the migrating eels were caught was on the Sawica River in the village of Janów, near Szczytno (NE Poland). The fish were caught with trap nets. After being caught, the fish were transported to the laboratory of the University of Warmia and Mazury, Olsztyn, Poland, where they were put into 1 m^3^ tanks, operating with closed water circulation [40]. All manipulation with fish was conducted in a state of anaesthesia (MS-222, Finquel, USA) at a dose of 300 mg L^−1^. After being anaesthetised, the fish were measured (TL; ±1.0 mm), weighed (±0.1 g), and labelled with individual PIT (passive integrated transponder) labels (Biomark, Boise, ID, USA). During the spawning migration, the eels change the environment from fresh water to salt water. Thus, such conditions had to be mapped in these studies. The fish stayed in the freshwater tank (for two weeks, at a water temperature of 12 °C) until the start of the experiment [32,33]. The initial water temperature was set at 12 °C and the photoperiod was 0 light/24 dark [33]. The fish were kept in total darkness (0.0–0.2 lx), because they migrate to spawning grounds under such conditions. Subsequently, the water was salted (Aqua Nova, Australia) over seven days to the level of 35‰. The water salinity was progressively increased, twice daily at a rate 5‰ per day [32]. During the experiment, salinity was maintained at the level of 32–35‰. After 20 days, the water was heated to 15 °C (±0.1 °C) as the optimal for eel female maturation under controlled conditions [41]. The circulation had controllable physicochemical conditions (temperature, photoperiod) and the system was equipped with a PolyGeser filter and UV lamps (2 × 36 W each). The oxygen level in the water was at least 6 ppm. The fish were not fed throughout the experiment because migrating eels do not feed exogenously.

### 2.2. Hormonal Stimulation

Hormonal stimulation (*n* = 20) was started after the desired water salinity level was achieved according to the method described by Nowosad et al. [33] and Kucharczyk et al. [41]. After being taken out of the tanks, the fish were put in a container with an anesthetic. Subsequently, they had an intraperitoneal injection of carp pituitary homogenate (CPH, Argent, USA) at 18 mg kg^−1^. The fish were stimulated at 5-day intervals for 20 weeks. For ovulated females, the procedure was longer for the next five weeks.

### 2.3. Sampling

The fish were divided into a control group (fish without hormone stimulation) and two groups with hormonally stimulated fish. Five females were collected shortly after catching as a blind (control—beginning group) sample of the cadmium level in muscle and ovary. Twenty maturing (after hormonal treatment) female European eels were subjected to a cadmium (Cd) content analysis. Before sampling, all the fish handling was conducted in a state of anesthesia (egg collection) or euthanasia (tissue sampling) using MS-222 (Finquell, Redmond, WA, USA). Sample tissues (muscle, ovary, and in some cases eggs) were taken after ten weeks of the experiment from freshly captured specimens (*n* = 13) and from females (which start to ovulate) after 15 weeks of hormonal stimulation (*n* = 7). Eggs were stripped manually and they (unfertilized) were analysed for cadmium content from seven females after spawning. The sample tissues were stored in an ultra-low temperature freezer (Sanyo, MDF-U32V; Japan) at −80 °C until analysis. Fish and ovaries were weighed (to calculate gonadosomatic index (GSI) (%): 100 × gonad weight (g)/fish weight (g)) or fish and stripping eggs (to calculate pseudo-gonadosomatic index (PGSI) (%): 100 × egg weight (g)/fish weight (g)).

### 2.4. Cadmium Determination

Twenty female European eels were analysed for cadmium (Cd) content in muscles, ovaries, and eggs and five from control groups for CD content in muscle and ovary. The Cd content was determined by flameless atomic spectrometry, with an electrothermal atomizer (GFAAS graphite oven) in a spectrometer (iCE 3000 SERIES–THERMO, Loughborough, UK), fitted with a GLITE data station, cathode lamps, and Zeeman background correction. Measurements were performed at the wavelengths of 288.8 nm. Each tissue was examined in at least two replicates, with three spectrometer readings for each sample. Limit of detection (LOD): 0.068 ppb, limit of quantification (LOQ): 0.20 ppb.

### 2.5. Dry Weight Analysis

Dry weight content in muscle and ovary samples was determined by the following method (e.g., [33]): approximately 1 g samples (±0.0001 g) in duplicates were initially dried at 65–70 °C in quartz tests, then dried to a constant weight at 105 °C for 1 h [33].

### 2.6. Protein Analysis

The protein content in muscle and ovary samples was determined following the method of Kjeldahl according to PN-75 A-04018 (e.g., [33,37]). The measurements were done in triplicates.

### 2.7. Fat Analysis

Fat (lipid) content in muscle and ovary samples was determined using the method described below (e.g., [6,33]). Approximately 1 g duplicate samples (±0.0001 g) were dried to a constant weight at 105 °C in glass sample tubes with frits and transferred to weighed beakers. The lipids from the fish muscles (without skin) were hot-extracted in three steps (extraction, rinsing, and drying) (E-816HE automatic extractor, BUCHI, Switzerland). The content of fat (%) was calculated according to the following formula: x = [(b − a) × 100]/c; where *a* = weight of flask (g), *b* = weight of flask with extracted fat (g), and *c* = weight of samples (g).

### 2.8. Statistical Analysis

The results are presented as means with standard error (mean ± SE). The statistical analysis was performed in Microsoft Excel and Statistica v. 13.1 (StatSoft Inc. 2016, Tulsa, OK, USA). Variance homogeneity and data normality were tested using the Shapiro–Wilk and Levene tests, respectively. The data were analysed using a non-parametric Kruskal–Wallis one-way analysis of variance (ANOVA; *p* < 0.05). The regression model was used to describe the relationships between fish weight and length and cadmium content in muscle and ovary, between cadmium content in muscle and ovary, and between protein and fat content and cadmium content in muscle and ovary.

## 3. Results

**Figure 1:**
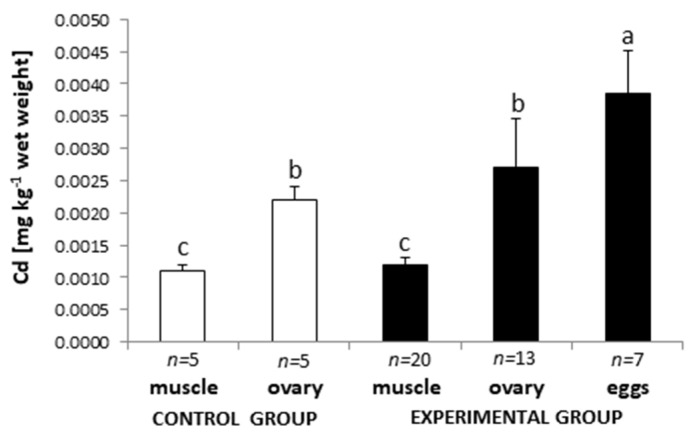
Cadmium content (mg kg^−1^ wet weight) in muscle (*n* = 5) and ovaries (*n* = 5) of hormonally untreated and in muscles (*n* = 20), ovaries (*n* = 13), and eggs (*n* = 7) of hormonally treated silver females European eel *Anguilla anguilla*. Data are presented as mean ± SD. Data marked with the same letter index (above the bars) do not differ statistically (*p* > 0.05).

The cadmium content in tissues of female European eel caught in the Baltic Sea Catchment Area is individually diversified (Figure 1). Statistically significant differences (*p* < 0.05) were observed between the mean cadmium content in muscles and ovaries and eggs (*p* < 0.05). The lowest mean cadmium level was found in muscles (0.0012 ± 0.0001 mg kg^−1^; *p* < 0.05; *n* = 20) and the highest was in the eels’ eggs (0.0038 ± 0.0007 mg kg^−1^; *n* = 13; *p* < 0.05; Figure 1).

**Figure 2:**
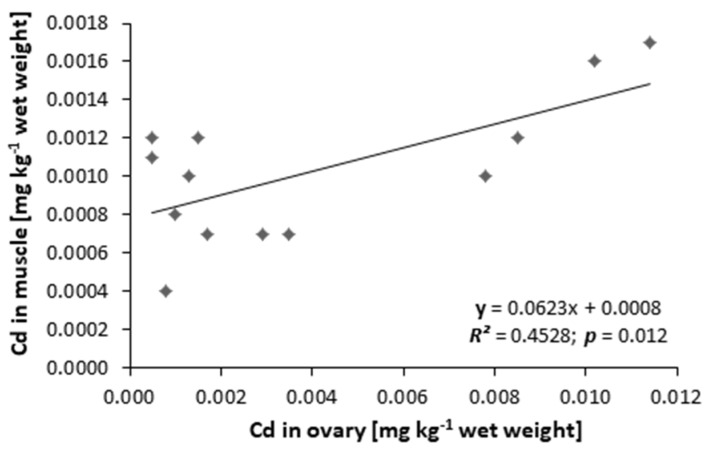
Relationship between cadmium content (mg kg^−1^ wet weight) in muscles and its content in ovaries of silver female European eel *Anguilla anguilla* (*n* = 13).

In the control group, the mean cadmium level was lower: 0.0011 ± 0.0001 mg kg^−1^, *n* = 5 and 0.0022 ± 0.0002 mg kg^−1^, *n* = 5 for muscles and ovaries, respectively, but no statistical differences between control and treated groups were found. No relationships were found between the weight and length of the tested female eels (non-hormonally or hormonally stimulated) and the level of cadmium in muscles, ovaries, and eggs. A close correlation was found between the cadmium content in muscles and ovaries in the same fish (*R* = 0.673; *p* = 0.0117; Figure 2).

**Figure 3:**
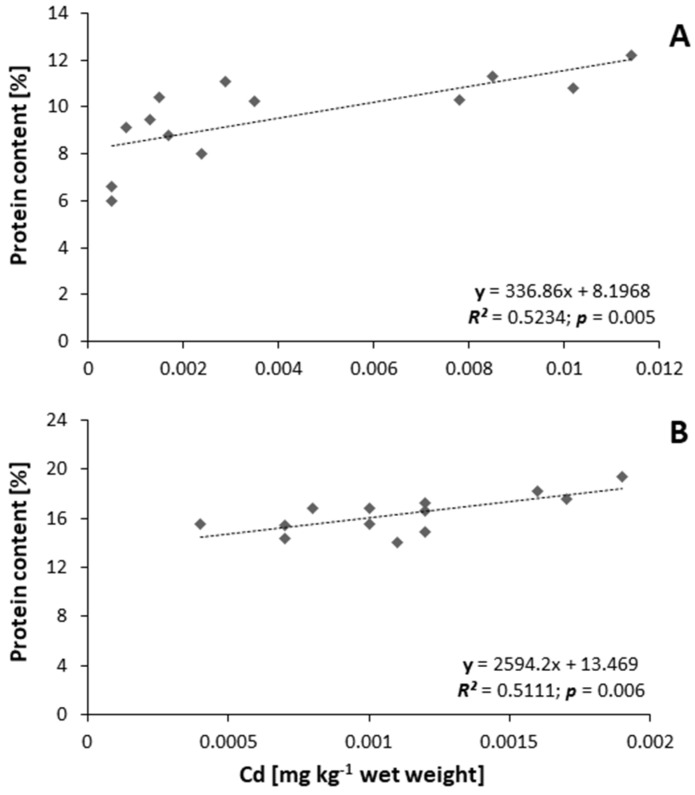
Relationship between percentage content of protein and content of cadmium in ovaries (**A**) and muscles (**B**) of silver European eel *Anguilla anguilla* (*n* = 13).

The water content in the tissues was slightly different. In muscle, this percentage was (mean ± SE) 58.55 ± 1.64%, while in the ovaries, it was 61.39 ± 1.21%. The fat content percentage (mean ± SE) was 26.28 ± 1.18 and 22.19 ± 1.21 for ovaries and muscles, respectively. Moreover, a positive correlation was observed between the percentage content of protein and cadmium content (mg kg^−1^ wet weight) in ovaries (*R*^2^ = 0.5111; *p* = 0.0052; Figure 3A) and in muscles (*R*^2^ = 0.5111; *p* = 0.0060; Figure 3B).

**Figure 4:**
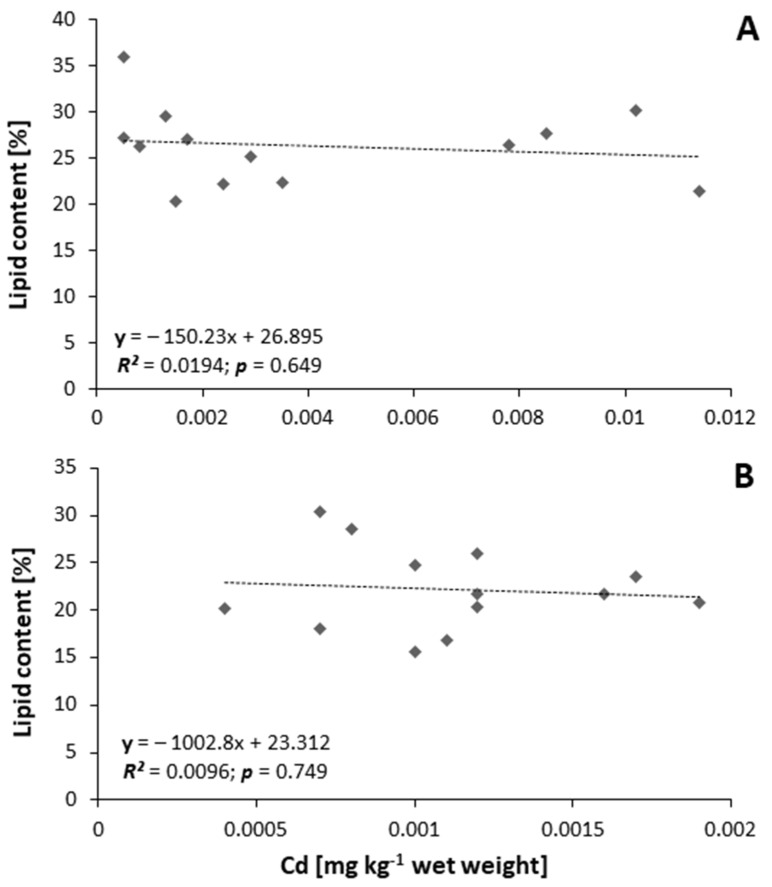
Relationship between percentage content of fat (lipids) and content of cadmium in ovaries (**A**) and muscles (**B**) of silver European eel *Anguilla anguilla* (*n* = 13).

The correlations between the percentage content of fat and cadmium content (mg kg^−1^ wet weight) in ovaries (*R*^2^ = 0.0194; *p* = 0.649; Figure 4A) and in muscles (*R*^2^ = 0.0096; *p* = 0.749; Figure 4B) were not significant. The GSI values (*p* < 0.05) for the control (beginning group) fish were 1.72–2.04% (*n* = 5; control group), and those for the hormonally stimulated fish after ten weeks were 13.21–18.46% (*n* = 13). The PGSI values for hormonally stimulated and ovulating fish were 20.22–30.08 (*n* = 7; at the end of the experiment).

## 4. Discussion

This is the first study of the cadmium content in female eel tissues including ovulated eggs. The dynamics of cadmium in muscle, gonads, and eggs during maturation under controlled conditions were also noted. It was found that the level of cadmium was higher in ovaries than in muscles, which may indicate its active transport from tissues to gonads. Unfortunately, the highest content of this heavy metal was observed in eggs (after ovulation), which, consequently, may have a negative impact on the fertilization process, embryonic development, and embryo survival rate.

In maturing female European eels, the content of water, protein, and fat in tissues (muscles and ovaries) in this study did not differ from the data provided by Nowosad et al. [33]. Importantly, in both cases, the research was carried out on fish from the same catching place of migrating eels. Heavy metals can infiltrate the body by two major routes: gills and the alimentary tract. Heavy metal concentration may be different in different fish organs or tissues [38,42]. The highest concentrations of Cd are observed in the kidneys (e.g., [13]) or in eggs (present study). The lowest concentration of this heavy metal was found in muscles [e.g., 8, present study]. The muscles of silver European eel females, as determined in this study, were found to contain 0.001 ± 0.000 mg kg^−1^ wet weight (range from 0.0004 to 0.0028), which is much less than in eels in Mar Menor (SE Spain): 0.002 ± 0.001 (nd-0.047) mg kg^−1^ wet weight [18] and Hutovo Blato (Bosnia and Herzegovina): 0.02 ± 0.003 (0.016–0.026) mg kg^−1^ wet weight [8]. The muscles of European eels caught in the waters of France were found to contain less cadmium by as much as 10 and 45 µg g^−1^ dry weight in the liver and kidneys, respectively [17]. The same tendency was noted by Has-Schön et al. [8] for eels from Bosnia and Herzegovina. Pierron et al. [31] demonstrated that eels exposed to cadmium pollution showed slower growth of body weight and lower effectiveness of lipid accumulation in muscles. This is important as eels stop feeding during migration and use lipids and proteins accumulated in tissues as a source of energy [33]. An insufficient amount of energy may result in their failure to reach the spawning site [40]. The studies conducted by Marsh-Matthews et al. [43] and by Kottmann et al. [44] showed that nutrients are transported from the mother to the embryo during the fish maturation process. If the transfer of nutrients is accompanied by a transfer of heavy metals, it poses a serious threat to the health of future offspring and their survival.

This study showed that eel eggs contain 1.4 times more cadmium than gonads and 3 times more than muscles in the same fish. This may be indicative of a considerable transfer of the element from the mother’s body to the ovaries and then to eggs. This is important because the gonadosomatic index (GSI) in female eels is under 2%, and it exceeds 30% before ovulation [31,33]. Therefore, a considerable increase in the ovary weight is accompanied by a considerable growth of the cadmium level (this study). Many morphological changes occur in the European eel body during the maturation process [32,33,38], with a consequent transfer of proteins and lipids to ovaries [33,45]. Freese et al. [38] demonstrated that the transfer of toxic metals from soft and hard tissues to the ovaries takes place during the maturation of female European eels. Similar observations on the maternal transfer of hazardous substances, including organic pollutions, dioxin, and heavy metals in female eels, were published by other authors [10,46,47]. Many hazardous compounds, including organic pollutants, dioxins, and some heavy metals, are transported from the tissues of the European eel, including muscles and bones, into the gonads and thus into the eggs. In some cases, gonadal Cd levels increased approximately seven times [47] compared with other tissues during fish maturation. This means that the European eel is a species extremely vulnerable to the accumulation of pollutants. This is because of the fact that the eel is a top predator and it is also a long-lived species, which means that it can accumulate pollutants in the tissue for a long time. In the present study, the level of cadmium in eggs was approximately 3.3 times higher than in muscles. According to many studies, including Freese et al. [38] and Belpaire et al. [47], the content of organic pollutants and heavy metals in eel tissues is extremely variable. Additionally, Belpaire et al. [47] reported that the level of Cd in muscle tissues in eels can range from less than 1 to 2472 μg kg^−1^ wet weight. This applies not only to fish from different locations, but also to a group caught in one place and at one time [8,37]. There may be many reasons for this phenomenon, for example, a different period of life spent in freshwater, and thus a different period of accumulation of pollutants in the eel’s body. Moreover, individual variability in the type of food consumed cannot be ruled out, which undoubtedly affects the chemical composition of the fish body [48]. In other fish species, such as roach *Rutilus rutilus*, perch *Perca fluviatilis*, chub *Squalius cephalus*, and sichel *Pelecus cultratus*, the pollution content may vary between populations, but is usually stable within one group [6,7,48,49]. The pollutants, however, have different levels in different tissues in fish. For example, much smaller amounts of mercury (Hg) were found in muscles than in ovaries of sichel [49] and European eel [37]. European eel eggs contained 8 and 78 times less mercury than in the ovaries and muscles, respectively. This may suggest that there is a barrier between muscles and eggs, which protects offspring from mercury transfer from the mother’s body [37]. However, the opposite trend was observed in the case of cadmium in this study. The reason for these differences is not yet known. In the case of Hg in several species, including eel, it was found that there is a barrier against Hg transport from the tissues to the ovaries and eggs [e.g., 37,49]. The lack of a similar protective mechanism in fish in the case of Cd may indicate that this pollution in waters appeared relatively recently, e.g., in the case of the development of heavy industry, and the fish have not yet managed to generate a defense mechanism. This study showed a positive correlation between cadmium content and protein content in muscles and ovaries of European eel, which may suggest a significant effect of proteins in cadmium transfer from the females’ tissues to eggs. A higher cadmium content in offspring from females exposed to cadmium compared with the control group was observed in two species of livebearer fish—western mosquitofish (*Gambusia affinis*) and least killifish (*Heterandria formosa*) [50]. The authors observed that the cadmium level in offspring decreased in each consecutive litter. Female fish exposed to cadmium may give birth to offspring (litter) earlier and the second litter may be delayed [50] or the reproduction process may be inhibited [51]. Cadmium can strongly stimulate the pituitary–gonadal–liver axis in maturing female eels and induce early and intensive vitellogenesis [52]. The process of vitellogenesis involves the formation of vitellogenin, which is transported in the blood to ovaries, where it is the main protein in the yolk [53]. Cadmium may be transported with vitellogenin to ovaries and subsequently to eggs. Therefore, cadmium may not only disrupt the hormonal balance, but it may also affect the quality and number of eggs. Embryos exposed to cadmium are likely to exhibit frequent birth defects and low survival rates [54,55,56].

## 5. Conclusions

Cadmium is one of the most hazardous heavy metals and is harmful to the health and reproduction of living organisms. Nutrients are transferred from the eel body to its gonads during the fish maturation process. Cadmium is probably transferred together with proteins. The muscles of European eels were found to contain three times less cadmium than eggs. This is a cause for concern, as cadmium, even in small amounts, can cause numerous birth defects in offspring.

## Data Availability

The data presented in this study are available on request from the corresponding author.
